# Multibranch convolutional neural network with contrastive representation learning for decoding same limb motor imagery tasks

**DOI:** 10.3389/fnhum.2022.1032724

**Published:** 2022-12-13

**Authors:** Chatrin Phunruangsakao, David Achanccaray, Shin-Ichi Izumi, Mitsuhiro Hayashibe

**Affiliations:** ^1^Neuro-Robotics Laboratory, Graduate School of Biomedical Engineering, Tohoku University, Sendai, Japan; ^2^Presence Media Research Group, Hiroshi Ishiguro Laboratory, Advanced Telecommunications Research Institute International, Kyoto, Japan; ^3^Department of Physical Medicine and Rehabilitation, Graduate School of Biomedical Engineering, Tohoku University, Sendai, Japan; ^4^Department of Robotics, Graduate School of Engineering, Tohoku University, Sendai, Japan

**Keywords:** brain-computer interface, ensemble learning, representation learning, motor-imagery, motor rehabilitation

## Abstract

**Introduction:**

Emerging deep learning approaches to decode motor imagery (MI) tasks have significantly boosted the performance of brain-computer interfaces. Although recent studies have produced satisfactory results in decoding MI tasks of different body parts, the classification of such tasks within the same limb remains challenging due to the activation of overlapping brain regions. A single deep learning model may be insufficient to effectively learn discriminative features among tasks.

**Methods:**

The present study proposes a framework to enhance the decoding of multiple hand-MI tasks from the same limb using a multi-branch convolutional neural network. The CNN framework utilizes feature extractors from established deep learning models, as well as contrastive representation learning, to derive meaningful feature representations for classification.

**Results:**

The experimental results suggest that the proposed method outperforms several state-of-the-art methods by obtaining a classification accuracy of 62.98% with six MI classes and 76.15 % with four MI classes on the Tohoku University MI-BCI and BCI Competition IV datasets IIa, respectively.

**Discussion:**

Despite requiring heavy data augmentation and multiple optimization steps, resulting in a relatively long training time, this scheme is still suitable for online use. However, the trade-of between the number of base learners, training time, prediction time, and system performance should be carefully considered.

## 1. Introduction

Strokes, a leading cause of death worldwide, occur when blood flow to the brain is restricted, preventing brain tissues from receiving a sufficient supply of oxygen (Armour et al., 2016). Most survivors experience paralysis—the permanent loss of muscle control. The power of the mu and beta oscillatory rhythms in the sensorimotor areas decreases and increases in response to motor events during the processes of motor imagery (MI) and real motor execution (Pfurtscheller and da Silva, 1999). These phenomena are known as event-related desynchronization (ERD) and event-related synchronization (ERS), respectively. ERD/ERS patterns are typically observed using electroencephalograms (EEG), which offer affordability, non-invasiveness, and high temporal resolution (Bereś, 2017). The MI-based brain-computer interface (MI-BCI) is a promising technology that provides a communication pathway to control computer applications and peripheral devices by translating motor intentions from EEG signals to computer commands (Padfield et al., 2019). This system allows users with motor impairments to manipulate orthoses and assistive robots simply by imagining physical motions (Venkatakrishnan et al., 2014; Bhattacharyya et al., 2016; He et al., 2018). The high elicitation of ERD during MI execution contributes to the motor recovery process (Pfurtscheller and Neuper, 2001; Chaudhary et al., 2015). Consequently, the MI-BCI has gained significant attention in the field of motor rehabilitation.

Although the field of MI-BCI has experienced significant developments over the past several decades, the general use of EEG-based BCI remains hindered by the poor spatial resolution of EEG, which limits the range of unique MI tasks that the system can distinguish (Yong and Menon, 2015). Other suboptimal characteristics of EEG, such as a low signal-to-noise ratio and high dimensionality, also represent major challenges in BCI (Rashid et al., 2020). Common MI tasks employed to build the system include hand, foot, and tongue imaging (Ang et al., 2012). The classification of such tasks by conventional machine learning algorithms has been relatively successful, as these tasks activate spatially well-separated regions of the motor cortex. However, they offer only one degree of freedom for orthotic control usage (Pfurtscheller et al., 2003). For practical purposes, the system must be able to decode a wider range of motions, particularly within the same limb. To date, only a handful of studies have examined the decoding of different MI tasks within the same limbs, and most have yet to produce acceptable results, as the tasks activate very close or overlapping regions of the brain (Plow et al., 2010).

Recent advancements in hardware and software have led to the development of increasingly sophisticated algorithms, such as deep learning (DL). DL applications have demonstrated significant improvements over classical machine-learning approaches across various domains (Goodfellow et al., 2015). DL algorithms automatically discover discriminative features from raw inputs, thus eliminating the need for handcrafted features and enabling end-to-end processing. Consequently, DL architectures -specifically convolutional neural networks (CNN) (Schirrmeister et al., 2017; Lawhern et al., 2018; Sakhavi et al., 2018)—are increasing in popularity as their performance outperforms that of traditional approaches. However, the scarcity of available training samples in BCI often leads to overfitting. As a result, DL in BCI can only yield marginal improvements over handcrafted methods (Phunruangsakao et al., 2022).

Ensemble learning is a method that employs multiple individual DL models, known as base learners, to build a single strong learner. This approach can enhance classification performance, increase robustness, and reduce overfitting compared to a typical DL model (Dietterich, 2000; Ganaie et al., 2021). Accordingly, this paper proposes a novel multi-branch CNN (MBCNN) to improve the classification of hand MI tasks (grasping, flexion, and extension). MBCNN assumes that each DL model can learn different discriminative features. Thus, the models compensate for each other's drawbacks. By concatenating deep features, the classifier can fully leverage informative feature representation, thereby improving its discriminating capabilities. However, the concatenated features may induce adverse effects, such as a slower training process and overfitting owing to an increase in redundant and irrelevant features (Yu and Liu, 2004). To address this issue, contrastive representation learning has been applied to MBCNN (MBCL) to group features together by class. In addition, an optimized weighted voting strategy with differential evolution (DE) is employed to validate whether model assemblage *via* feature concatenation improves overall system performance compared to prediction voting.

## 2. Related work

The loss of motor functions due to nervous system disorders and injuries is a long-standing medical concern. Over the past several decades, MI-BCI has been anticipated to restore motor functions by allowing computerized devices to be controlled by brain signals. However, the practical applications of this approach are severely limited, as current systems have relatively low degrees of freedom, classification performance, and robustness for robotic control. Several methods, including ensemble learning, have recently been proposed to address these issues.

A base learner refers to an individual learner, feature extractor, or classification model that can be assembled *via* ensemble learning to form a single strong learner. Ensemble learning often yields higher classification accuracy than traditional DL models (Dietterich, 2000; Ganaie et al., 2021). Most existing ensemble learning approaches for MI-BCI can be categorized as ensemble classification or feature combinations. The underlying concept of ensemble classification is to combine predictions from multiple classifiers to improve the generalizability or robustness of a single-base classifier. Feature combinations comprise sets of features extracted from the same raw data using different extraction methods. The combination of different features can boost classifier performance by providing stronger discriminative descriptors to the classifier.

One approach to ensemble classification is the transformation of a multiclass classification problem to a multiple binary classification problem. This can be achieved using a one-vs-one (OvO) or one-vs-rest (OvR) strategy. Liao et al. (2014) used an OvO strategy to assemble 10 binary classifiers corresponding to 10 pairs of finger movements that use movement-related spectral changes as features. They achieved an average classification rate of 77.1% when a support vector machine was employed as the classifier. The study by Vuckovic and Sepulveda (2008) adopted a similar approach to classifying pairs of extension, flexion, pronation, and supination, and obtained a decoding accuracy as high as 80% in certain subjects. Geng et al. (2008) applied OvR to reduce the number of classifiers corresponding to four mental tasks, whereas Jeunet et al. (2015) used combined shrinkage linear discriminant analysis (Lotte and Guan, 2010) to decode MI tasks and explore relationships between the users' control performance, personality, cognitive profiles, and neurophysiological markers.

In contrast to the methods previously mentioned, ensemble classification by meta-learning does not require the addition of new base learners as more classes are added. Instead, the metalearning algorithm combines predictions from multiple base learners trained on the same dataset to enhance the ensemble model's generalizability. Silva et al. (2017) demonstrated that ensemble multi-layer perceptron (MLP) models outperform single MLPs under an appropriate training scheme. Ramos et al. (2017) explored the voting ensemble to make the final prediction based on the sum of predictions made by the base learners. They combined the outputs of 11 different classification algorithms and compared the performance of the voting ensemble *via* majority voting and weighted majority voting. The results show that voting weights optimized by the genetic algorithm produced the optimal results. Bashashati et al. (2016) built an ensemble model from classifiers trained with different hyper-parameters (frequency bands, channels, and time intervals), automatically tuned by Bayesian optimization. This approach yielded results similar to those obtained by state-of-the-art methods. The multi-branch 3D CNN developed by Zhao et al. (2019) utilizes three CNNs with different filter sizes to extract a wider variety of features, wherein the CNNs' softmax outputs are combined to obtain the final predictions. An insufficient number of base learners may cause instability in the ensemble classifier, whereas an excessive number of base learners introduces high computational complexity. This trade-off must be carefully evaluated, as a low prediction time and high classification accuracy are important for the system to be practical (Ruta and Gabrys, 2005).

C2CM (Sakhavi et al., 2018) uses a feature combination by modifying the filter bank common spatial pattern (FBCSP) (Ang et al., 2012), and combining the envelope representation with a CNN to aid pattern recognition within the input. Riyad et al. (2020) added 4 different branches to EEGNet (Lawhern et al., 2018), enabling it to derive meaningful feature representation. The approach developed by Amin et al. (2019) concatenates deep features from several CNNs with different architectures to improve MI classification accuracy. Li et al. (2019) utilized a channel-projection mixed-scale CNN to account for the spatial dependencies and varying temporal information of EEG input. TS-SEFFNet (Li et al., 2021) incorporates squeeze-and-excitation feature fusion to map temporal and multispectral features onto a representation space. A study by Özdenizci et al. (2018) exploited EEG and electromyography signals to decode complex hand gestures using hierarchical graphical models.

Although feature combinations can yield more informative features that aid classification, a degree of feature learning must be incorporated to eliminate redundant and irrelevant features that may confuse the classifier. Özdenizci and Erdoğmuş (2019) utilized information theoretic feature transformation learning to reduce the confounding effects of heuristic feature ranking and selection caused by dimensionality reduction approaches, such as common spatial patterns. Ma et al. (2022) developed a time-distributed attention network that contains class- and band-attention submodules. NeuroGrasp (Cho et al., 2021) integrates a CNN and bidirectional long short-term memory for feature extraction. The features were subsequently adapted using convolutional SiamNet with contrastive loss. Although these methods have demonstrated great potential for decoding complex tasks within the same limb, their classification accuracy remains relatively insufficient for practical use.

## 3. Methodology

**Figure 1:**
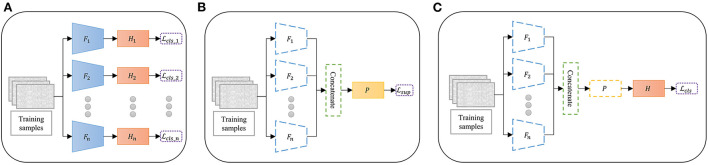
Overview of the proposed multibranch convolutional neural network with contrastive representation learning *via* sequential optimization. **(A)** Pretraining: The base learners are separately pretrained on the same dataset. **(B)** Contrastive representation learning: The features of the pre-trained extractors are concatenated and optimized. **(C)** Fine-tuning: The classifier is trained on the projected feature. Dashed lines indicate the frozen model, *F* is the feature extractor, *H* is the classifier, *P* is the feature projector, *L*_*cls*_ is the cross entropy loss function, and *L*_*sup*_ is the supervised contrastive loss function.

The proposed multi-branch CNN with contrastive representation learning (MBCL) was trained by sequential optimization with a sliding window to augment the data. The optimization comprises three sequential steps: pretraining, contrastive representation learning, and fine-tuning. During pretraining (Figure 1A), multiple base learners were initialized and individually optimized on the same training samples to address the same multiclass classification problem using Equation (3). The base learner is a typical DL architecture that includes a feature extractor and a classifier, wherein the feature extractor is assumed to learn a unique representation. The concatenation of these features can aid the classification process by providing more informative, distinctive, and independent features. Three base learners were employed during the experiment: ShallowConvNet, DeepConvNet, and EEGNet. The base learner architecture is summarized in Table 1.

**Table 1:** Architecture and parameters of each module, and ELU is exponential linear unit.

**Module**	**Layer**	**Filter**	**Tohoku MI-BCI**		**BCI dataset IIa**		**Activation**
			**Kernel**	**Output dim**	**Kernel**	**Output dim**	
ShallowConvNet (Schirrmeister et al., 2017)	Input	-	-	(16, 128, 1)	-	(22, 1,000, 1)	-
	Conv2D	40	(1, 13)	(16, 116, 40)	(1, 26)	(22, 976, 40)	-
	Conv2D	40	(16, 1)	(1, 116, 40)	(16, 1)	(1, 976, 40)	-
	BatchNormalization	-	-	(1, 116, 40)	-	(1, 976, 40)	ELU
	AveragePooling2D	-	-	(1, 12, 40)	-	(1, 61, 40)	Log
	Dropout	-	-	(1, 12, 40)	-	(1, 61, 40)	-
	Flatten	-	-	(480)	-	(2,440)	-
DeepConvNet (Schirrmeister et al., 2017)	Input	-	-	(16, 128, 1)	-	(22, 1,000, 1)	-
	Conv2D	25	(1, 2)	(16, 124, 25)	(1, 3)	(22, 991, 25)	Linear
	Conv2D	25	(16, 1)	(1, 124, 25)	(22, 1)	(1, 991, 25)	Linear
	BatchNormalization	-	-	(1, 124, 25)	-	(1, 991, 25)	ELU
	MaxPooling2D	-	-	(1, 62, 25)	-	(1, 330, 25)	-
	Dropout	-	-	(1, 62, 25)	-	(1, 330, 25)	-
	ConvBlock	50	(1,5)	(1, 29, 50)	(1,10)	(1, 107, 50)	ELU
	ConvBlock	100	(1,5)	(1, 12, 100)	(1,10)	(1, 32, 100)	ELU
	ConvBlock	200	(1,5)	(1, 4, 200)	(1,10)	(1, 7, 200)	ELU
	Flatten	-	-	(800)	-	(1,400)	-
EEGNet (Lawhern et al., 2018)	Input	-	-	(16, 128, 1)		(22, 1,000, 1)	-
	Conv2D	8	(1,64)	(16, 128, 8)	(1,128)	(22, 1,000, 8)	-
	BatchNormalization	-	-	(16, 128, 8)	-	(22, 1,000, 8)	ELU
	DepthwiseConv2D	16	(16,1)	(1, 128, 16)	(22,1)	(1, 1,000, 16)	-
	BatchNormalization	-	-	(1, 128, 16)	-	(1, 1,000, 16)	ELU
	AveragePooling2D	-	(1,4)	(1, 32, 16)	(1,8)	(1, 125, 16)	-
	Dropout	-	-	(1, 32, 16)	-	(1, 125, 16)	-
	SeparableConv2D	16	(1,16)	(1, 32, 16)	(1,32)	(1, 125, 16)	-
	BatchNormalization	-	-	(1, 32, 16)	-	(1, 125, 16)	ELU
	AveragePooling2D	-	(1,8)	(1, 4, 16)	(1,16)	(1, 7, 16)	-
	Dropout	-	-	(1, 4, 16)	-	(1, 7, 16)	-
	Flatten	-	-	(64)	-	(112)	-
Feature projector	Input	-	-	(1,344)	-	(3,952)	-
	Dense	-	16	(16)	16	(16)	ELU
Classifier	Input	-	-	(16)	-	(16)	-
	Dense	-	6	(6)	4	(4)	Softmax

Owing to the intra-subject variability and poor spatial resolution of EEG signals, the deep feature mapping of equivalent MI tasks may be too sparse, whereas that of different MI tasks may be too compact. Moreover, deep feature representation from different feature extractors may introduce irrelevant and redundant features that can skew classifier performance. To address these issues, contrastive representation learning was conducted, wherein each classifier was detached from its base learner to allow feature concatenation. Subsequently, a feature projector was added for contrastive representation learning (Figure 1B). The feature extractors were frozen in this step, as an excessive number of trainable parameters in the extractors may cause overfitting when mapping the contrastive features. The feature projector was optimized *via* supervised contrastive loss, as shown in Equations (1), (2).

After the feature representation was optimized, the classifier head was added on top of the feature projector (Figure 1C). The layers below the classifier were frozen when the classifier was fine-tuned on the projected features using Equation (3). Finally, an end-to-end model was built and prepared for classification.

### 3.1. Network architecture

#### 3.1.1. Feature extractor

Feature extraction is a process wherein relevant information or characteristics are derived such that the raw input can be easily interpreted (Azlan and Low, 2014). Therefore, it is crucial to increase the effectiveness of the classifiers. Raw EEG signals are usually assumed to carry spatio-temporal information; therefore, they cannot be treated as a special type of images (Lotte et al., 2007). FBCSP is among the most widespread feature extraction algorithms that learn spatio-temporal features from multiple subbands of EEG signals (Ang et al., 2012). Although FBCSP has successfully demonstrated its strength in extracting discriminative features from EEG, it is sensitive to noise and artifacts. Recently, many studies have introduced DL approaches that mimic the functionality of FBCSP.

Inspired by the success of CNNs in computer vision, Schirrmeister et al. (2017) developed DeepConvNet, which can extract a wide range of features. Its architecture comprises four convolutional-max-pooling blocks. The first block was specifically designed to handle a large number of EEG channels by sequentially applying temporal convolutions, spatial filtering, and max-pooling with a linear activation function. The remaining three blocks employ standard convolutional-max-pooling (ConvBlock) with ELU activation for feature extraction. The authors further proposed ShallowConvNet. The first two layers of ShallowConvNet perform the temporal and spatial filtering, which resemble the bandpass and spatial filtering steps in FBCSP. Likewise, batch normalization, average pooling, and logarithmic activation resemble the log-variance computation in FBCSP. Unlike FBCSP, however, ShallowConvNet allows all steps to be jointly optimized by combining them into a single end-to-end model. Lawhern et al. (2018) introduced a compact CNN architecture called EEGNet, which uses depth-wise and separable convolutional layers to reduce the trainable parameters, thus avoiding overfitting.

The present study employed ShallowConvNet, DeepConvNet, and EEGNet as feature extractors for MBCNN, MBCL, and DE. Each model's architecture is summarized in Table 1. Because the sampling frequency of Dataset IIa is double that of the Tohoku MI-BCI dataset, the kernel lengths of the feature extractor are different.

#### 3.1.2. Feature projector

Owing to the intra-subject variability and overlapping activation of brain regions generated by MI tasks, training a classification model with only traditional cross-entropy loss may cause suboptimal generalizability in the model. The core idea of contrastive representation learning is to project feature representations such that samples from the same class are grouped together, whereas samples from different classes are projected further apart (Khac et al., 2020). To achieve this, a feature projector is employed to learn contrastive representations from the deep convolutional features produced by feature extractors. The projector comprises a single 16-unit dense layer with exponential linear unit (ELU) activation, which was empirically found to produce the optimal results. While contrastive learning can be applied in both supervised and unsupervised settings, a supervised setting is more suited for MI-BCI because it allows for the full utilization of labeled samples. Conversely, an unsupervised setting requires a large number of training samples, which are generally not available in MI-BCI.

During contrastive representation learning, a feature projector is trained using supervised contrastive loss (Khosla et al., 2020). The loss function is formulated as follows:


(1) $$\begin{array}{l} L_{sup}=\underset{i\in I}{\sum }L_{sup}^{i} \end{array}$$

(2) $$\begin{array}{l} \underset{i\in I}{\sum }L_{sup}^{i}=\underset{i\in I}{\sum }\frac{-1}{P\left(i\right)}\underset{p\in P\left(i\right)}{\sum }\log\frac{\exp\left(z_{i}\cdot z_{p}/\tau \right)}{\sum_{a\in A\left(i\right)}\exp\left(z_{i}\cdot z_{a}/\tau \right)} \end{array}$$

Where *i* ∈ *I* ≡ {1…*N*} is the index of the sample in the mini-batch, *A*(*i*) ≡ *I*\{*i*} is the index without *i*, *z* is the projected feature, τ is the temperature constant, and $P\left(i\right)\equiv \left\{p\in A\left(i\right):{\tilde{y}}_{p}={\tilde{y}}_{i}\right\}$ is the set of indices for all positive samples without *i*.

Temperature plays a significant role in determining the robustness of a classifier. While a smaller temperature tends to produce better results, the numerical instability makes it more difficult to train. To optimize the results, the temperature in the present study was empirically set at 0.05. Data augmentation is also essential in contrastive learning, as it increases the number of negative and positive samples. Accordingly, a sliding window was employed to simulate data augmentation.

#### 3.1.3. Classifier

A single dense layer with softmax activation is used to decode the motor intention from the feature representation. The shallow architecture of the classifier allows features to be processed directly, thus minimizing overfitting. The classifier outputs the probability of the sample belonging to each class and is optimized using cross-entropy loss, represented by the sum of the negative logarithms of the predicted probabilities of each class:


(3) $$\begin{array}{l} L_{cls}=-𝔼\overset{cls}{\underset{k=1}{\sum }}{𝟙}_{\left(y==k\right)}\log\left(H\left(z\right)\right) \end{array}$$

where 𝟙 is an indicator function dependent on the condition that *y* = =*k*, *H* is the classifier, and *z* is the projected feature.

## 4. Experiment

To verify the benefits of contrastive representation learning, the performance of MBCL was compared with that of MBCNN, wherein contrastive representation learning and the feature projector were disabled. Because MBCNN did not utilize the feature projector, its training steps only encompassed base learner pretraining and classifier fine-tuning. Because feature concatenation may induce feature redundancy, MBCNN and MBCL were additionally compared with an optimized weighted voting ensemble strategy using differential evolution (DE), as described in Section 4.2. To ensure a fair comparison, MBCNN, MBCL, and DE used the same feature extractor and classifier.

All models were implemented in the TensorFlow library using the Keras API and trained on an Intel Core i7 CPU with an NVIDIA GeForce GTX 1070 GPU. The models were fitted using the Adam optimizer with a learning rate of 1*e*−3, batch size of 32, and dropout probability of 0.5. The training was terminated when cross-entropy loss (Equation 3) had stopped improving for 10 epochs.

### 4.1. Dataset description and preprocessing

#### 4.1.1. Tohoku University MI-BCI dataset

The dataset (Achanccaray et al., 2021) was acquired from the Neuro-Robotics Laboratory, Tohoku University. It includes EEG data from 18 able-bodied subjects (15 males and 3 females; 17 right-handed and 1 left-handed; aged between 19 and 39 years) who performed 20 trials of 3 motor imagery tasks (grasping, flexion, and extension) on each hand. Brain activity was recorded with a 16-channel g.USBamp (g.tec Medical Engineering GMBH) amplifier with a sampling frequency of 512 Hz. Wet active electrodes were placed at AF3, AF4, FC3, FCz, FC4, C3, Cz, C4, T7, T8, CP3, CPz, CP4, Pz, O1, and O2, in accordance with the 10–20 international system. During data acquisition, subjects were instructed to minimize their head and eye movements.

**Figure 2:**
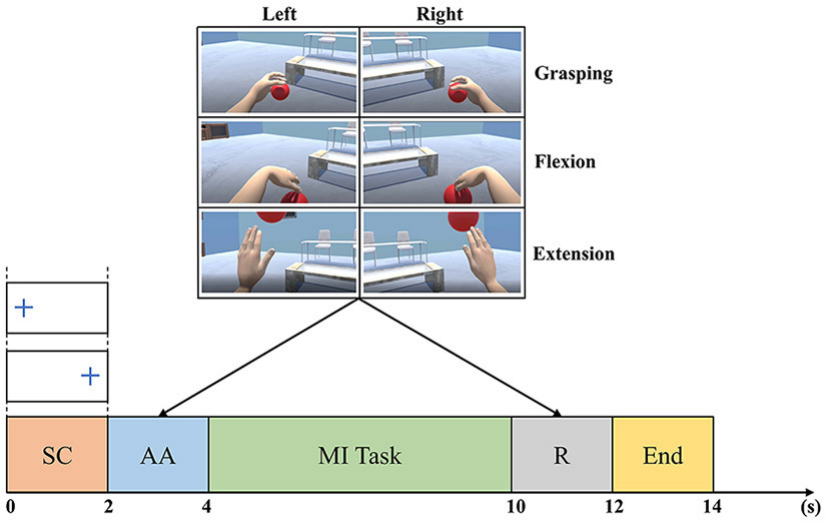
Timing of the Tohoku University MI-BCI dataset signal acquisition. The trial timeline is a sequence of a side cue (SC), an animation (AA) of the MI task requested, a MI task performing, a visual reinforcement (R), and an end cue (Achanccaray et al., 2021).

Figure 2 shows the signal acquisition timing scheme. A fixation cross is shown during [0, 2]s of each trial to indicate the side of the MI task. Consequently, an animation of the randomly chosen MI task is played during [2, 4]s. Next, the subject is asked to repeatedly perform the requested MI task for 6 s. Then, the subject is given a visual reinforcement for 2 s. Finally, a blue line is shown on the screen to signal the end of the trial.

The 6-s MI trials were downsampled by a factor of 4 and filtered using an eighth-order Butterworth bandpass filter with a cut-off frequency of 0.5 and 30 Hz and a fourth-order 50 Hz notch filter, artifact removal and standardization were not performed, following the preprocessing steps described in the original paper (Achanccaray and Hayashibe, 2020) for fair comparison. The dataset was divided into training and test sets using 5 repetitions of stratified 5-fold cross-validation with different randomization for each repetition, i.e., the experiments done on this dataset were evaluated 25 times on different sets of training and test samples with no data leakage.

There have been several attempts to decode multiple hand-MI tasks, but most of the studies focused only on tasks from either left or right hand (Edelman et al., 2015; Yong and Menon, 2015; Achanccaray and Hayashibe, 2020; Chu et al., 2020). Hence, this experiment aims to expand the applicability of MI-BCI system by decoding multiple MI tasks from both hands. Furthermore, the MI tasks exemplified by this dataset are highly demanding for motor rehabilitation, as upper-body paralysis is the most common. Therefore, this dataset was selected for the experiment.

The training times for MBCNN and MBCL under sequential optimization and sliding windowing on this dataset were 279.41 ± 4.34s and 296.36 ± 8.58s, respectively.

#### 4.1.2. BCI competition IV dataset IIa

The dataset (Tangermann et al., 2012) comprises 22-channel EEG samples from 9 subjects performing left/right hand, foot, and tongue MI movements (72 trials for each task). All signals were sampled at 250 Hz. Subjects were asked to perform an MI task for 4 s.

The samples were filtered using a third-order Butterworth bandpass filter with cutoff frequencies of 4 and 38 Hz, and later standardized using electrode-wise exponential moving standardization to reduce noise (Gramfort et al., 2013), following the procedure described in Schirrmeister et al. (2017) for fair comparison. The standardization is formulated as follows:


(4) $$\begin{array}{l} x_{k}^{\prime }=\frac{x_{k}-\mu_{k}}{\sqrt{\sigma_{k}^{2}}} \end{array}$$

Where $x_{k}^{\prime }$ and *x*_*k*_ are standardized signal and raw signal at time *k*, respectively. μ_*k*_ and $\sigma_{k}^{2}$ denote exponential moving average and variance which are calculated by:


(5) $$\begin{array}{l} \begin{array}{c} \mu_{k}=\left(1-\alpha \right)x_{k}+\alpha \mu_{k-1}\\ \sigma_{k}^{2}=\left(1-\alpha \right){\left(x_{k}-\mu_{k}\right)}^{2}+\alpha \sigma_{k-1}^{2} \end{array} \end{array}$$

Where α is the decay factor, set to 0.999. At the beginning of each epoch, μ_0_ and $\sigma_{0}^{2}$ were set as the mean and variance, respectively, of each electrode. The dataset was split in accordance with the competition guideline (Ang et al., 2012), where two EEG recording sessions were used for training and testing, respectively. The experiment on this dataset was repeated ten times to reduce bias.

This dataset, which is commonly employed as a benchmark, was selected to verify that the proposed scheme can effectively decode MI tasks not only within the same limb but also among different limbs.

The training times for MBCNN and MBCL under sequential optimization and sliding windowing on this dataset were 103.77 ± 9.90s and 117.94 ± 13.04s, respectively.

#### 4.1.3. Sliding window

The lack of training data in MI-BCI often leads to overfitting. Data augmentation is a process that mitigates this issue by generating new samples *via* small modifications to the original samples. Schirrmeister et al. (2017) demonstrated that the use of sliding windows to create multiple crops from each trial improves the model's generalizability and robustness while reducing overfitting. Likewise, a sliding window was used to augment the samples in this study.

The trials on the Tohoku University MI-BCI dataset were reshaped using a 1-s window with a 0.1-s stride. The first window starts at the onset time, and the last window ends at the trial time, in accordance with the original procedure (Achanccaray et al., 2021). This resulted in a total of 53 windows per trial.

Schirrmeister et al. (2017) used a 2-s sliding window to augment the samples in BCI Dataset IIa, wherein the first window starts at 0.5 s before onset time, and the last window ends with the trial, resulting in 625 windows per trial. However, in this study, the samples in BCI Dataset IIa were reshaped using a 4-s window with a 0.1-s stride to reduce the computational load.

Note that there was no data leakage between the training and test sets.

### 4.2. Optimized weighted voting ensemble strategy

Theoretically, an increase in the number of training features improves the DL model's discriminating power. However, training an ensemble model on excessive training samples has adverse effects, such as a slower training process and overfitting owing to feature redundancy (Yu and Liu, 2004). The voting ensemble strategy combines predictions from multiple models to obtain a final prediction. In contrast to the feature combination approach, this strategy does not share feature representations among the models and reduces coupling between different MI tasks, resulting in less feature redundancy and higher robustness (Duan et al., 2014; Subasi and Mian Qaisar, 2021). Therefore, it was used for comparison with the MBCNN and MBCL models in this experiment.

Because some base learners are assumed to be more reliable than others, this study proposes an optimized weighted voting ensemble strategy, wherein each base learner is assigned a different weight or contribution to the final prediction. The softmax outputs from the base learners were multiplied by the assigned weights, and the class with the highest summed probability was selected for the final prediction. The weights were optimized using a differential evolution (DE) (Storn and Price, 1997), whose objective function is to maximize the classification accuracy within the training dataset.

**Figure 3:**
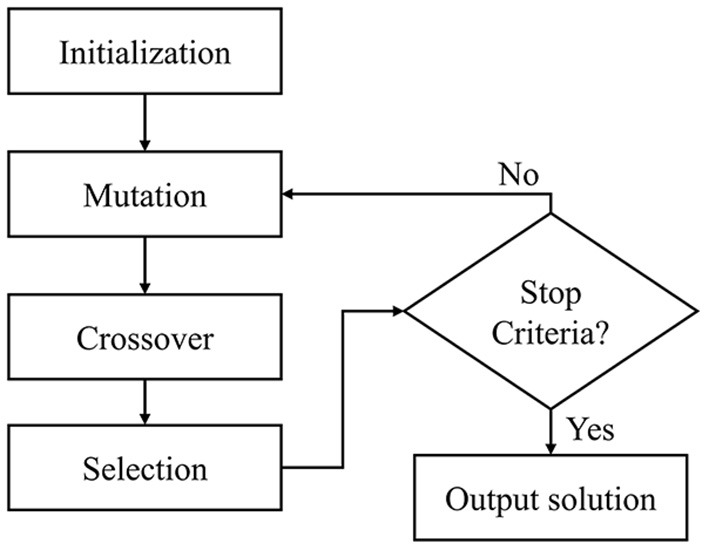
Flow chart of differential evolution algorithm.

DE is a population-based optimization method that iteratively selects the optimal candidate through an evolutionary process. As shown in Figure 3, the algorithm consists of four steps: initialization, mutation, crossover, and selection. It begins with a random population initialization of possible solutions. During each iteration, the population vectors (parents) undergo mutation and crossover to produce a large variety of candidate solutions (offspring). The selection step replaces the parents with the offspring, yielding a lower objective function value. It was found that 100 generations, 50 populations, [0.5, 1] mutation probability, and 0.7 crossover probability produced the best results during the empirical experiment. Accordingly, those parameters were assigned for the weight optimization.

## 5. Result and discussion

The experimental results on the Tohoku University dataset (Achanccaray et al., 2021) with six MI classes are presented in terms of classification accuracy, Cohen's kappa value, *p*-value, and average prediction time. The kappa value was used to assess the inter-rater reliability, or likelihood that the results were generated by chance. It ranges between –1 and 1, where –1 represents total disagreement, 0 indicates no agreement, and 1 denotes perfect agreement. The values are calculated as follows:


(6) $$\begin{array}{l} \kappa =\frac{p_{0}-p_{e}}{1-p_{e}} \end{array}$$

Where *p*_0_ is the classification accuracy and *p*_*e*_ is the random classification accuracy. A statistical test was performed to observe the significance between MBCL and the alternative methods. The normality of the classification accuracy was tested using the Shapiro-Wilk (S-W) test, where a *p*-value exceeding 0.05 suggests normality. Because the test's results indicate that the accuracy does not exhibit a normal distribution, a Mann-Whitney *U*-test was employed to calculate the statistical significance, where a *p* < 0.05 implies a significant statistical difference. One major drawback of the multi-branch architecture is its high computational complexity. Therefore, the average prediction time must be assessed to determine whether the prediction model is suitable for online applications. This was calculated by averaging the time taken by the model to predict all test samples.

Because there is no released code for comparable ensemble learning approaches, these results were solely compared with those obtained by the base learners. Therefore, the proposed methods were subsequently evaluated on BCI Dataset IIa with four MI tasks to further validate their performance. These results were compared with those obtained by several state-of-the-art approaches, including the BCI competition IV winner (FBCSP; Ang et al., 2012), feature combination approaches (CP-MixedNet; Li et al., 2019, TS-SEFFNet; Li et al., 2021, Incep-EEGNet; Riyad et al., 2020, and C2CM; Sakhavi et al., 2018), and ensemble classification methods (3D CNN; Zhao et al., 2019 and BO; Bashashati et al., 2016). Because the comparative results on BCI Dataset IIa were obtained from original papers and re-implementations, only the classification accuracy and kappa value were used as evaluation metrics. Results from the Tohoku University and BCI IIa datasets are presented in Tables 2, 3, respectively. The highlighted results indicate the optimal values for each case.

**Table 2:** Performance comparison of different approaches on six MI classes Tohoku University MI-BCI dataset, where highlighted results indicate the best values for each case.

**Subject**	**ShallowConvNet** **(Schirrmeister et al., 2017)**	**DeepConvNet** **(Schirrmeister et al., 2017)**	**EEGNet** **(Lawhern et al., 2018)**	**DE**	**MBCNN**	**MBCL**
T01	42.42	42.75	33.50	44.09	44.05	**44.21**
T02	69.83	66.88	67.57	71.78	70.48	**72.95**
T03	80.50	76.65	78.24	81.58	80.90	**83.05**
T04	69.34	63.3	72.45	72.85	71.33	**75.28**
T05	53.17	58.21	48.75	58.22	60.06	**61.05**
T06	67.98	65.11	59.55	69.47	71.55	**73.11**
T07	71.54	69.21	70.30	70.94	70.87	**72.39**
T08	48.76	47.47	49.05	49.95	50.12	**51.49**
T09	54.46	52.21	51.17	54.87	55.80	**56.46**
T10	71.23	61.74	61.62	72.32	70.26	**73.28**
T11	59.59	56.52	56.92	60.01	59.92	**62.34**
T12	48.75	40.85	48.85	48.50	46.48	**48.97**
T13	65.45	61.71	66.27	64.14	64.83	**66.26**
T14	58.87	54.08	47.50	59.05	58.16	**60.97**
T15	70.07	69.92	62.59	72.04	**72.55**	71.75
T16	71.25	67.23	70.18	72.14	71.42	**73.55**
T17	44.39	42.48	37.68	46.23	47.97	**50.38**
T18	34.09	32.74	27.63	33.82	33.46	**36.15**
Average	60.09	57.17	56.10	61.22	61.12	**62.98**
Kappa	0.5043	0.4671	0.4552	0.5179	0.5176	**0.5410**
*p*-value	0.0018	≪0.05	≪0.05	0.0597	0.0448	-
Time (ms/sample)	2.4013	2.6254	**2.3501**	2.9345	3.1824	3.3186

**Table 3:** Performance comparison of different approaches on four MI classes BCI competition IV dataset IIa, where highlighted results indicate the best values for each case.

	**Subject**									**Average**	**Kappa**
	**A01**	**A02**	**A03**	**A04**	**A05**	**A06**	**A07**	**A08**	**A09**		
FBCSP (Ang et al., 2012)	76.00	56.50	81.25	61.00	55.00	45.25	82.75	81.25	70.75	67.75	0.6000
3D CNN (Zhao et al., 2019)	77.40	60.14	82.93	**92.29**	**75.84**	68.99	76.04	76.85	84.66	75.01	0.6440
CP-MixedNet (Li et al., 2019)	74.65	53.47	73.26	70.14	67.36	48.96	74.31	72.92	69.44	67.17	0.5620
TS-SEFFNet (Li et al., 2021)	82.29	49.79	87.57	71.74	70.83	63.50	82.92	81.53	81.94	74.71	0.6630
TSSM (Xie et al., 2017)	80.00	58.70	86.30	68.20	60.30	59.20	84.40	**84.00**	**89.60**	74.52	0.5930
BO (Bashashati et al., 2016)	82.12	44.86	86.60	66.28	48.72	53.30	72.64	82.33	76.35	68.13	0.5751
Incep-EEGNet (Riyad et al., 2020)	78.47	52.78	89.93	66.67	61.11	**60.42**	90.62	82.29	84.37	74.07	0.6540
C2CM (Sakhavi et al., 2018)	**87.50**	**65.28**	**90.28**	66.67	62.50	45.49	89.58	83.33	79.51	74.46	0.6595
DE	82.69	45.78	89.76	69.00	62.31	52.89	**94.42**	80.10	83.20	73.35	0.6447
MBCNN	80.06	46.30	88.91	71.16	65.70	54.40	92.46	79.51	82.74	73.47	0.6429
MBCL	82.74	52.85	88.20	77.27	67.92	58.15	94.37	80.51	83.33	**76.15**	**0.6820**

The results from Tohoku University dataset reveals that the ensemble techniques (DE, MBCNN, and MBCL) significantly outperform the non-ensemble techniques (ShallowConvNet, DeepConvNet, and EEGNet) in all cases. This is mainly due to the capability of the base learners to compensate for one another's weaknesses, as some base learners may be able to learn discriminative features other base learners cannot, and provide informative features to the classifier. Upon comparing the statistical significance between MBCL and other approaches, it is found that there is a statistically significant between all pairs (*p* < 0.05). Additionally, DE and MBCNN are compared. The main difference between these methods lies in how the base learners are assembled. MBCNN concatenates the features from each base learner and feeds them into the classifier, whereas DE utilizes a voting strategy to optimize the prediction of the base learners. Despite the potential harmful effects that redundant and irrelevant features may have on the classifier in MBCNN, the results show that the classification accuracy of DE and MBCNN from both dataset are closely matched. Therefore, the negative effects of such features are negligible. MBCNN also exhibited superior performance over several feature combination approaches and DE outperformed some ensemble classification methods on BCI dataset IIa.

The concatenated features from feature combination approaches, including MBCNN, are unlikely to be optimized for subsequent classification. To improve the discriminative power of the classifier, MBCL employs contrastive representation learning, which leverages annotated samples to accommodate intra-subject non-stationarity and enhance feature representation. This allows the features to be more discriminative and minimizes the intra-class distance while maximizing the inter-class distance. Consequently, the proposed MBCL approach produced the optimal decoding performance for both datasets.

Although the average classification accuracy of MBCL was the highest on BCI Dataset IIa, it did not produce the highest accuracy in any individual subject. The most plausible explanation is that the hyperparameters for training were not optimized for this dataset as they were set empirically. Specifically, the temperature constant (τ) in Equations (1), (2) notably contributes to the feature mapping for each subject. By applying contrastive representation learning without proper value of τ, the resulting feature mappings were unlikely to be optimal for the classifier. This issue can be mitigated by performing cross-validation.

**Figure 4:**
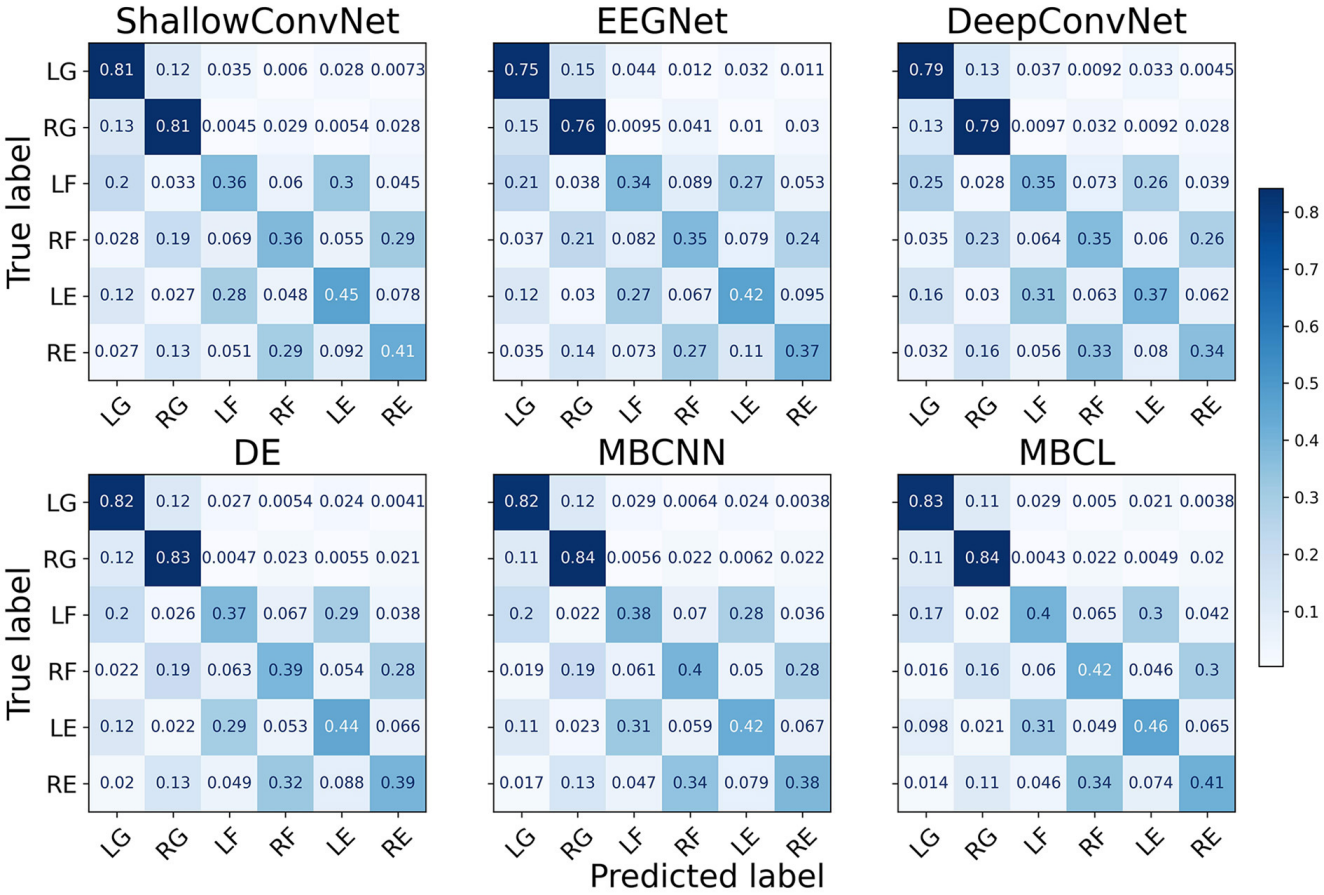
Confusion matrix of different approaches on Tohoku University MI-BCI dataset. The classes are abbreviated as follows; LG, Left Grasping; RG, Right Grasping; LF, Left Flexion; RF, Right Flexion; LE, Left Extension; RE, Right Extension.

**Figure 5:**
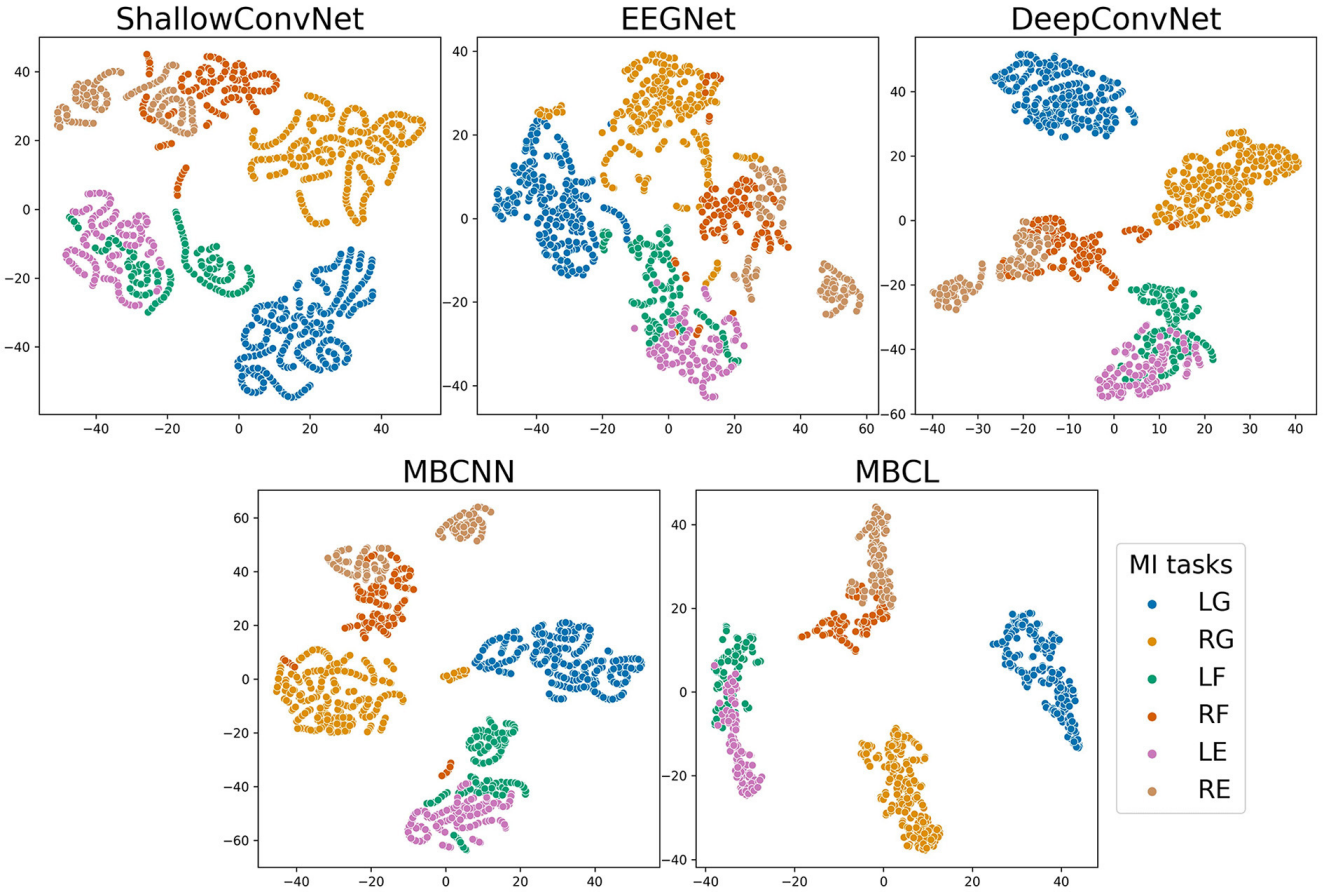
t-SNE plot of feature embedding on subject 3 of Tohoku University MI-BCI dataset. The classes are abbreviated as follows; LG, Left Grasping; RG, Right Grasping; LF, Left Flexion; RF, Right Flexion; LE, Left Extension; RE, Right Extension.

As illustrated by the confusion matrices (Figure 4), the predictions generated by different classification models and ensemble techniques can enhance prediction performance for all classes. The flexion and extension tasks activate overlapping brain regions related to wrist movements, whereas grasping activates finger-movement-related brain regions (Sanes et al., 1995). Although all models performed adequately well on grasping, this was not the case for flexion and extension. Regardless of enhanced classification performance, ensemble techniques still have difficulties classifying MI tasks within the same limb, as the base learners cannot effectively discriminate among them. The t-distributed stochastic neighbor embedding (van der Maaten and Hinton, 2008) (t-SNE) plot in Figure 5 depicts the feature distribution mapped using different approaches on Subject 3 of the Tohoku University dataset. This confirms that MBCL can map features of the same class more compactly, and features of different classes more distantly, than other methods, as it was trained under contrastive representation learning. Despite the additional training step, the confusion between flexion and extension shown by the MBCL was still high. Specifically, the accuracy of flexion and extension was half that of grasping. This illustrates the fact that the discriminative performance of an ensemble model depends heavily on the base learners. Furthermore, the dataset provides 16-channel EEG signals, which may fail to effectively capture important information in overlapping brain regions, leading to poor discriminability among tasks within the same limb.

Recent studies have shown that graph-based approaches are able to produce higher classification accuracy than CNN since they are more effective at capturing long-term temporal dependencies and intricate functional brain connectivity among channels (Stefano Filho et al., 2018; Zhang et al., 2020; Demir et al., 2021). CNN's basic presumption that electrodes are equally spaced apart, akin to picture pixels, prevents it from exploring discriminative EEG representations (Zhong et al., 2022). Although applying a 2D convolutions to each EEG trial may mitigate the issue, the flattening of 3D representations for classification may cause information loss. Therefore, adopting graph neural network or transformer, rather than CNN, as base learners could potentially increase the performance of the proposed method.

In addition, because DL models usually exhibit high complexity, they require sufficiently large datasets for training to generate accurate predictions (Adadi, 2021). DL models trained on insufficient data exhibit high variance and error during testing, as they learn noise or misleading patterns from the training samples. This phenomenon is known as overfitting. Because the number of training samples in BCI is severely limited by inconvenient and time-consuming calibration sessions (Rashid et al., 2020), overfitting is a common problem in DL-based BCI systems.

To mitigate the issue of overfitting, sequential optimization and sliding windowing were incorporated into the proposed method. Sequential optimization refers to the process in which each module is sequentially and separately optimized to reduce the number of trainable parameters during each step, as explained in Section 3. In contrast, joint optimization simultaneously optimizes all modules *via* multi-objective optimization, as in a typical DL training scheme. The final objective function is formulated as follows:


(7) $$\begin{array}{l} L=L^{cls}+L^{sup} \end{array}$$

Additionally, sliding windowing was employed to increase the number of samples by augmenting the data. This approach divides a trial into several crops, as described in Section 4.1.3, whereas trial-wise windowing uses the entire trial for training and testing.

Tables 4, 5 show the performance and statistical significance of the proposed methods under different training schemes, respectively. It is apparent that joint optimization and trial-wise windowing yielded the lowest performance due to overfitting. The training schemes that incorporated either sequential optimization or sliding windowing exhibited enhanced performance. However, the results on BCI Dataset IIa were marginally improved and exhibited no statistical significance, with the exception of the comparison between trail-wise windowing with sequential optimization and joint optimization on MBCL. It is possible that joint optimization yields higher performance than sequential optimization for sufficiently large datasets, as it jointly optimizes all modules and objective functions. However, for smaller datasets, such as those used in BCI, sequential optimization is recommended. The new samples that were created *via* sliding windowing enhanced the overall training process by reducing the possibility of overfitting while improving the effectiveness of contrastive representation learning. When both sequential optimization and sliding windowing were applied, performance improved significantly.

**Table 4:** Performance comparison of methods under different training schemes.

		**Sequential optimization**				**Joint optimization**			
		**Sliding window**		**Trial-wise**		**Sliding window**		**Trial-wise**	
**Dataset**	**Method**	**Average**	**Kappa**	**Average**	**Kappa**	**Average**	**Kappa**	**Average**	**Kappa**
Tohoku MI-BCI	DE	61.22	0.5179	49.94	0.3819	-			
	MBCNN	61.12	0.5176	46.24	0.3341	54.58	0.4436	20.58	0.0001
	MBCL	62.98	0.5410	39.33	0.2458	56.57	0.4679	21.58	0.0095
BCI dataset IIa	DE	73.35	0.6447	65.24	0.5366	-			
	MBCNN	73.47	0.6429	66.13	0.5484	65.05	0.5347	65.04	0.5339
	MBCL	76.15	0.6820	66.77	0.5569	65.04	0.5338	61.59	0.4879

**Table 5:** Statistical significance (*p*-value) of methods under different training schemes, where underlined values indicate no significant statistical difference.

		**Sequential optimization vs. joint optimization**		**Sliding window vs. trial-wise**	
**Dataset**	**Method**	**Sliding window**	**Trial-wise**	**Sequential optimization**	**Joint optimization**
Tohoku MI-BCI	DE	-		≪0.05	-
	MBCNN	≪0.05	≪0.05	≪0.05	≪0.05
	MBCL	≪0.05	≪0.05	≪0.05	≪0.05
BCI dataset IIa	DE	-		0.0002	-
	MBCNN	≪0.05	0.6719	0.0035	0.9316
	MBCL	≪0.05	0.0104	≪0.05	0.0819

Table 6 illustrates the performance of the proposed methods from empirical experiments when different numbers of base learners were utilized. The methods with three base learners (ShallowConvNet, DeepConvNet, and EEGNet) were compared with those with two and four base learners where EEGNet were removed and an additional ShallowConvNet were added, respectively. It is evident that incorporating fewer base learners decreased the computational time. In contrast, more base learners did not always yield the best classification results. From the empirical experiment, two base learners were unlikely to provide optimal feature representation, whereas four base learners tended to overfit the data. Therefore, only three base learners were employed in this study. This emphasizes the importance of choosing the appropriate number of base learners as trade-off between computation time and classification accuracy is critical.

**Table 6:** Performance comparison of different approaches on six MI classes Tohoku University MI-BCI dataset with different number of base learners.

	**Base learners**	**Average**	**Kappa**	***p*** **-value**	**Time (ms/sample)**
DE	2	60.74	0.5120	0.0140	2.2811
	3	61.22	0.5179	0.0597	2.9345
	4	61.23	0.5175	0.0611	3.1459
MBCNN	2	58.59	0.4852	≪0.05	2.8076
	3	61.15	0.5176	0.0448	3.1561
	4	59.81	0.5015	0.0002	3.3917
MBCL	2	61.16	0.5180	0.0252	2.8685
	3	62.98	0.5410	-	3.2803
	4	62.01	0.5293	0.2215	3.3419

## 6. Conclusion

This study proposes a framework for multi-branch CNN with contrastive representation learning to integrate base learners, thus improving BCI performance in decoding multiple MI tasks. The framework achieves this by concatenating features from the base learners to produce more discriminative and informative classification features. However, the concatenated features may contain redundant or irrelevant information. To mitigate this, MBCL employs contrastive representation learning to map representations, such that similar samples are grouped together, whereas distinct samples are set far apart. Furthermore, the approach was compared with a differential evolution method that uses an optimized voting strategy and does not share feature representations among base learners. The results demonstrate that the proposed framework outperformed all single classification models and voting strategies. However, the improved performance was strictly limited by the base learners' ability to distinguish discriminative features, and the framework's scalability is limited. Although the increase in base learners improves system performance, there is a trade-off in terms of prediction and training time; therefore, the number of base learners must be carefully considered. The proposed framework also relies on heavy data augmentation and sequential optimization, which may further prolong training time.

## Data availability statement

The original contributions presented in the study are included in the article, further inquiries can be directed to the corresponding author.

## Author contributions

CP, DA, and MH conceived the research design. CP implemented and analyzed the results and wrote the manuscript. DA performed the data recording with participants. All authors reviewed the manuscript.
